# Do BKPyV genomic features underlie clinical divergence between kidney and hematopoietic transplant recipients?

**DOI:** 10.1080/21505594.2026.2679815

**Published:** 2026-06-01

**Authors:** Aurélien Aubry, Baptiste Demey, Virginie Morel, Véronique Descamps, Ophélie Fourdinier, Maud Salmona, Louison Collet, François Helle, Etienne Brochot

**Affiliations:** a Virology Department, Centre Hospitalier Universitaire Amiens-Picardie, Amiens, France; b Infectious Agents, Resistance and Chemotherapy Research Unit, AGIR UR4294 Université de Picardie Jules Verne, Amiens, France; c Nephrology Dialysis and Transplantation Department, Centre Hospitalier Universitaire Amiens-Picardie, Amiens, France; d Virology Department, Hopital Saint-Louis, Paris, France

**Keywords:** BK polyomavirus, kidney transplantation, hematopoietic stem cell transplantation, nephropathy, hemorrhagic cystitis, whole genome sequencing

## Abstract

BK polyomavirus (BKPyV) persists in the renourinary tract of most adults and can replicate under immunosuppression. In kidney transplant recipients (KTR), it may cause BKPyV-associated nephropathy (BKPyVAN), while in hematopoietic stem cell transplant recipients (HSCT), it is more often linked to hemorrhagic cystitis (HC). These clinical differences are generally attributed to the type of graft and immunosuppressive regimen. However, viral factors such as genotype or mutations might also influence tissue tropism and pathogenesis. This study aimed to compare the virological features of BKPyV between KTR and HSCT recipients and to explore possible associations with clinical manifestations. This retrospective study included 101 transplanted patients (66 KTR, 35 HSCT) at Amiens-Picardie University Hospital (France) between 2019 and 2023, with at least one episode of BKPyV DNAuria during post-allograft follow-up. Viral genotyping was performed by Sanger sequencing, while NGS (Next-generation Sequencing) provided complete coding genome sequences for 51 patients. Genotype distribution was similar in both groups, with Ib2 as the most frequent subtype. No genotype or mutation was associated with a specific graft type or complication, except for the small t antigen gene, which appeared to be more frequently mutated in KTRs. Viral replication occurred earlier and at higher levels in HSCT patients (mean peak DNAuria: 9.3 log_1__0_ vs 7.4 log_1__0_ copies/mL in KTR; *p* < 0.0001). In KTRs, patients with presumptive BKPyVAN were significantly older than those with asymptomatic replication. These findings suggest that viral genetic determinants play a lesser role in BKPyV replication and its clinical consequences compared to host-related factors.

## Introduction

The polyomavirus (BKPyV) is ubiquitous, with adult prevalence approaching 100%, and persists in a latent state in the renal and bladder epithelia [1]. In cases of immunosuppression, it can replicate and induce clinical manifestations. It is a well-established cause of BKPyV-associated nephropathy (BKPyVAN) in kidney transplant recipients (KTR) and hemorrhagic cystitis (HC) in hematopoietic stem cell transplant (HSCT) recipients. These two clinical presentations differ in presentation and pathophysiology. The immunosuppressive context differs between the two types of transplantation: KTRs typically receive long-term maintenance immunosuppression, which generally involves the combination of a calcineurin inhibitor (tacrolimus or ciclosporin) with an antiproliferative agent (mycophenolate mofetil) and/or corticosteroids [2]. This is preceded by induction therapy with anti – IL-2 receptor antibodies or antithymocyte globulin. In contrast, HSCT recipients receive a myeloablative or reduced-intensity conditioning regimen before transplantation, followed by graft-versus-host disease prophylaxis, usually based on a calcineurin inhibitor combined with methotrexate or mycophenolate mofetil, which is progressively reduced and then stopped according to clinical course [3].

In HSCT recipients, BKPyV-related HC typically occurs between 2 and 8 weeks post-transplant, with an increased incidence in cases of myeloablative conditioning [4,5]. Its pathophysiology remains hypothetical and seems to result from a combination of mechanisms, including urothelial lesions induced by myeloablative treatments on the urothelium, viral replication facilitated by immunosuppression, and immune damage mediated by donor T lymphocytes during immune reconstitution [5,6]. T immunity therefore has an ambivalent role, as it may contribute to urothelial lesions, although it is also essential for viral clearance [6–8].

In KTR, the renal graft may contain latent BKPyV, unknown to the recipient’s immune system. This context, combined with prolonged administration of immunosuppressive drugs, favors viral replication. BKPyV replication is then responsible for lytic destruction of the graft’s tubular epithelial cells. This lysis can lead to interstitial inflammation, followed by fibrosis and tubular atrophy, which can result in progressive loss of renal function. This histological and clinical picture corresponds to BKPyVAN [9].

Although the clinical divergence observed between KTR and HSCT recipients underlines the importance of the immunological context in pathogenesis, it seems important to keep in mind that BKPyV is not a homogeneous entity. Its circular double-stranded DNA genome encodes early regulatory proteins (large and small T antigens – Tag and tAg) and late proteins including the structural capsid proteins VP1–VP3 (Viral Protein 1–3) and the agnoprotein (Agno), separated by a non-coding control region (NCCR). This virus presents genetic diversity through the existence of several distinct genotypes (I to IV), themselves divided into subtypes, as well as significant genomic variations such as Single Nucleotide Polymorphisms (SNPs), notably in the BC loop of VP1 [10], and rearrangements or SNPs in the NCCR [11,12]. These genetic characteristics appear to modulate the viral replication capacity and tropism of BKPyV. In addition to factors related to the host and immunosuppression, certain viral characteristics could therefore play a role in the pathophysiology of BKPyVAN and HC.

On the one hand, the clinical forms classically found may not be explained solely by the type of transplant, as shown by certain case reports. Two HSCT recipients presented with histologically confirmed BKPyVAN and DNAemia exceeding 6 log_10_ copies/mL, one at 7 months and the other at 5 years post-HSCT [13,14]. Conversely, one KTR developed HC one month after transplantation, without bacterial infection or renal involvement, but with significant BKPyV DNAuria [15].

On the other hand, most epidemiological studies of BKPyV genotyping have been carried out in a single type of transplant recipient, either kidney or hematopoietic stem cell, or without distinguishing between these two distinct populations [16], which limits comparisons. Nevertheless, genotype Ia appears to be rare in studies about KTR, which is not always true in HSCT, where it is predominant in certain cohorts (in the US [17] and in Paris [18]). These data point to a possible link between viral genotype and the type of clinical disease.

The main aim of this study was to compare the virological characteristics of BKPyV (genotypes, mutations) between two cohorts: a KTR cohort with or without BKPyVAN, and a cohort of HSCT recipients with or without HC. To minimize epidemiological bias due to geographical variability, both cohorts were recruited from the same center, during the same period. The secondary objective is to evaluate the existence of potential associations between certain virological characteristics and the clinical manifestations observed.

## Materials and methods

### Cohort

This retrospective study used data and residual samples collected during routine care, with no additional sampling performed. All patients who underwent HSCT or kidney transplantation at Amiens-Picardie University Hospital (France) between January 2019 and December 2023, and who had at least one positive BKPyV PCR during post-transplant follow-up, were included. The exclusion criteria were insufficient residual sample volume for study purposes and opposition to the use of data for biomedical research.

This study was conducted in accordance with the MR-004 reference methodology (French Data Protection Authority, CNIL), which requires that patients are informed about the use of their data for research purposes; written consent was not required. Institutional approval was obtained from the Amiens University Hospital Research and Innovation Department (DRCI), under registration number PI2022-843–0079. The study was conducted in accordance with the principles of the Declaration of Helsinki.

Each cohort is subdivided into two groups. In the HSCT recipient group, patients are classified according to the presence or absence of BKPyV HC, as recommended by the ECIL (European Conference on Infections in Leukemia) [5]: BKPyV HC is defined by the triad of urinary functional signs, macroscopic hematuria, and BKPyV DNAuria greater than 7 log_10_ copies/mL. KTRs are divided according to the presence or absence of presumptive BKPyV nephropathy, defined by the presence of at least one plasma PCR higher than 4 log_10_ copies/mL, according to the 2024 criteria [19].

### Molecular biology

PCR detection of BKPyV was carried out prospectively, at the request of clinical departments, as part of post-transplant follow-up. Systematic screening was performed in KTR as recommended [19], whereas targeted testing was performed in HSCT recipients in the presence of clinical symptoms [20]. Urine samples showing BKPyV DNAuria for which a sufficient residual quantity was available were used for the retrospective study analyses. Before retrospective analysis, urine samples were stored at −80°C to ensure molecular stability. For the purpose of the study, viral DNA was extracted using the QIAmp Viral RNA Mini Kit and QIAcube automated system (Qiagen, Germany), according to the manufacturer’s recommendations.

Two sequencing approaches were used. First, genotype determination was performed in the first BKPyV-positive PCR sample of each patient, using PCR amplification of the BK typing and grouping region (BKTGR) using primers BKV-S1920 and BKV-A2159, followed by Sanger sequencing, as described by Morel *et al*. [21].

Coding region sequencing was performed using next-generation sequencing (NGS), after amplification and preparation of the libraries using the DeepChek® Assay Whole Genome BKV Genotyping kit (ABL DIAGNOSTICS S.A., Woippy, France). Samples were pooled at a final concentration of 80 pM and sequenced on the ISeq100 system (Illumina, San Diego, USA).

Bioinformatics analysis was performed using Galaxy. Reads were first filtered using Sickle, retaining only those with a quality score above Q30 and a minimum length of 50 bases. Alignment was performed with BWA-MEM using the Dunlop genome as a reference (GenBank accession: V01108.1), excluding the NCCR region, which was considered not to be representative of the wild-type. Duplicates were removed using MarkDuplicates, consensus sequences were generated using Ivar consensus, variants were identified using Varscan, and functional annotation was performed with SnpEff.

The consensus sequences were aligned with sequences available on GenBank of known genotype and with the MAFFT tool, then the phylogenetic tree was constructed with IQTree and visualized with iTOL v6. In cases where the inferred genotype did not match the results previously obtained by Sanger sequencing, the sequence was excluded from genotype analysis. The VESPA tool (Los Alamos National Laboratory) was used to search for nucleotide patterns potentially associated with a given clinical group. Finally, each patient sequence was realigned to a genotype-specific consensus sequence, constructed from available GenBank entries (Ia: 156 sequences, Ib1: 247, Ib2: 236, II: 27, IVc1: 46, IVc2: 67), in order to call variants independently of polymorphisms associated with the genotype.

### Statistical analysis

All statistical analyses were performed using R software (version 4.3.2) and GraphPad Prism (version 8.0, GraphPad Software, San Diego, CA, USA). Categorical variables were compared using the Chi-squared test and Fisher-exact test. For continuous variables, normality was assessed using the Shapiro – Wilk test. If the data followed a normal distribution and showed homoscedasticity, comparisons were made using a two-tailed Student’s t-test; otherwise, the non-parametric Mann – Whitney U test was applied. Results were considered statistically significant for *p-values* < 0.05.

## Results

### Cohort characteristics and BKPyV replication kinetics

**Figure 1.**
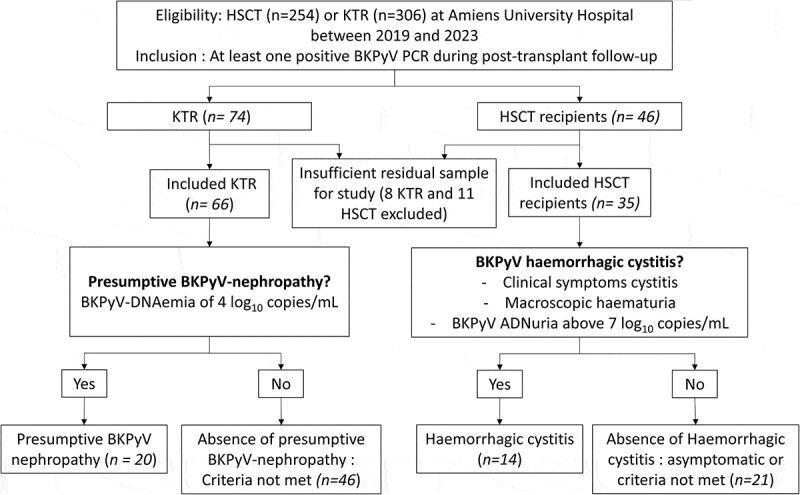
Flowchart of patient inclusion in the study.

A total of 101 patients were included in the study: 35 HSCT recipients, including 14 with HC, and 66 KTRs, of whom 20 had presumptive BKPyVAN. Patient characteristics are summarized in the flowchart (Figure 1) and Table 1. No significant difference was observed in the distribution of BKPyV genotypes between the two cohorts, with a homogeneous distribution across both HSCT and KTR groups.

**Table 1.** Clinical characteristics of the cohort. Quantitative data as mean (standard error of mean – SEM); qualitative data as n (%). For quantitative variables without normal distribution (Mann-Whitney test), the median [IQR – Interquartile range] is also provided.

		KTR (*n* = 66)		HSCT (*n* = 35)	*p-values*
Age (years)		54.35 (1.527) Med = 55.67 [45.12–62.66]		51.90 (3.017) Med = 57.29 [35.67–66.76]	0.9462^a^
Sex	M		48 (72.7)	23 (65.7)	0.4630^b^
	F		18 (27.3)	12 (34.3)	
Genotype	Ib1		10 (16.9)	5 (15.6)	0.9673^b^
	Ib2		34 (57.6)	20(62.5)	
	IV		10 (16.9)	5 (15.6)	
	Ia/II		5 (8.5)	2 (6.3)	
	Unknown		7	3	
Time from transplant to BKPyV PCR positivity (days)		94.7 (14.4) Med = 38.5 [31.25–118.25]		31.7 (6.2) Med = 31 [12–43]	**0.0007**^**a**^
BKPyV DNAuria peak (log_10_ copies/mL)		7.4 (0.26) Med = 7.45 [6.28–9.04]		9.3 (0.20) Med = 9.6 [8.80–10.13]	**<0.0001**^**a**^
BKPyV DNAemia peak (log_10_ copies/mL)		1.69 (0.21)		1.41 (0.25)	0.3983^c^

^a^Mann – Whitney U test (non-normal distribution); ^b^Chi-square test; ^c^Student’s t-test (normal distribution).

**Figure 2.**
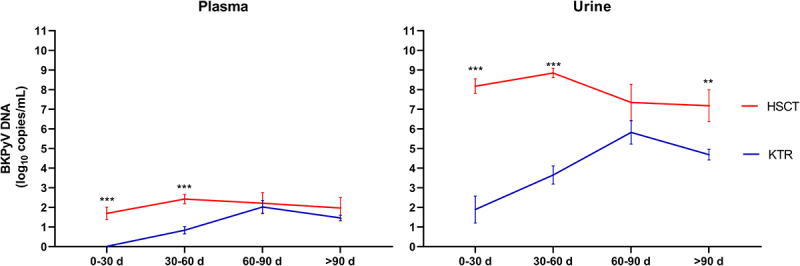
Kinetics of BKPyV replication in urine and plasma in HSCT and KTR recipients over time post-transplantation (d = day). Each point represents the mean ± SEM (standard error of the mean). *** = *p* < 0.001; ** = *p* < 0.01.

In contrast, viral replication kinetics differed markedly between the cohorts, as shown in the Figure 2. BKPyV replication occurred significantly earlier in HSCT recipients, with a first positive PCR detected at a median of 31 days post-transplant, compared to 38.5 days in KTRs (Mann – Whitney U test, *p* = 0.0007). Moreover, significantly higher viral DNA loads were observed in both urine and plasma during the early post-transplant period in HSCT patients (t-tests; 0–30 days: 1.89 vs 8.17 log_10_ copies/mL in urine, and 0.01 vs 1.69 in plasma; 30–60 days: 3.65 vs 8.84 log_10_ copies/mL in urine and 0.83 vs 2.42 in plasma; all *p < 0.0001*). In addition, the peak BKPyV DNAuria was significantly higher in HSCT recipients, with a median of 9.6 log_10_ copies/mL compared to 7.45 log_10_ copies/mL in KTRs (Table 1; Mann – Whitney U test, *p-value < 0.0001*). This difference was not observed for peak BKPyV DNAemia between HSCT and KTR.

### Sequencing analysis

**Figure 3.**
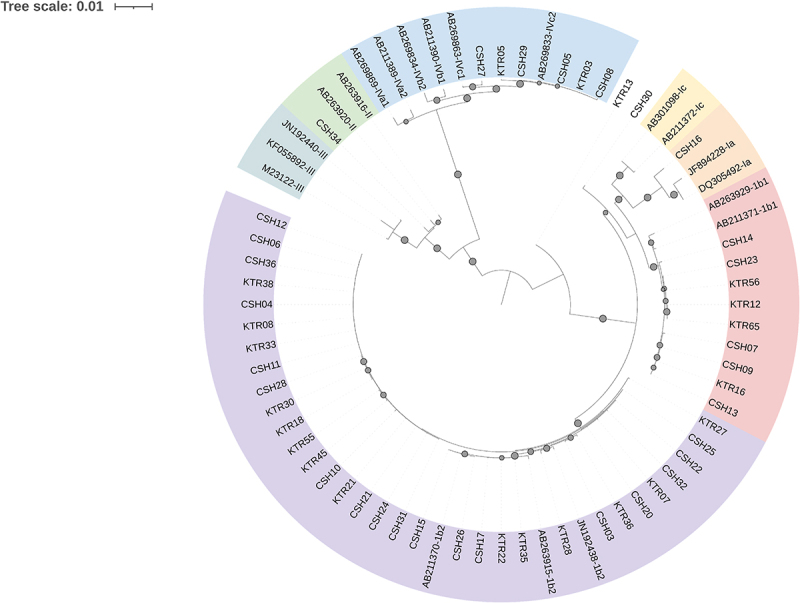
Phylogenetic tree based on consensus sequences (excluding the NCCR), aligned using MAFFT and constructed with IQ-TREE. Tree visualization was performed with iTOL v6. Only bootstrap values > 80 are shown (gray circles). Genotypes were assigned based on clustering with GenBank reference sequences and are color-coded accordingly. Patient sequences are labeled as “KTR” (kidney transplant recipient) or “CSH” (hematopoietic stem cell transplant recipient), followed by a patient-specific number. KTR13 and CSH30 were excluded from genotype analysis due to uncertain genotype assignment.

A subset of the cohort underwent BKPyV coding region sequencing using NGS, as described in the Materials and Methods section. For 30 HSCT recipients and 21 KTRs, the entire coding region was analyzed after alignment with the Dunlop reference sequence. The sequences have been deposited in GenBank under accession numbers PV856407 to PV856457. The phylogenetic tree shown in Figure 3 reveals no apparent clustering of sequences by clinical group, suggesting that the viral sequence is not associated with the type of transplant in which it replicates.

**Figure 4.**
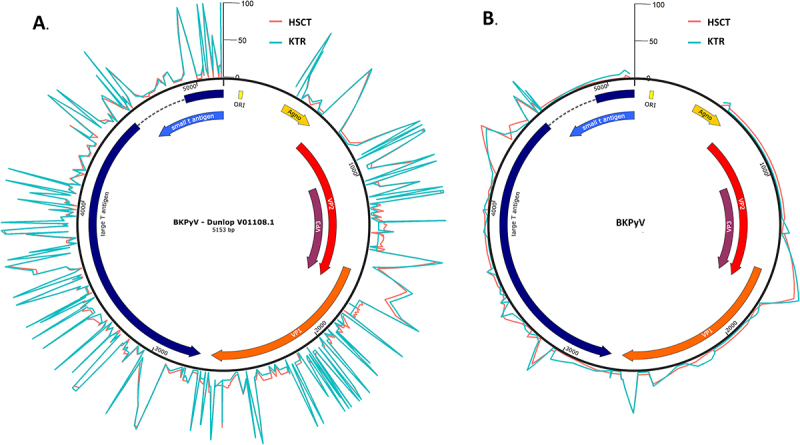
Representation of the BKPyV Dunlop genome (visualized with SnapGene) surrounded by two curves indicating the mutation frequencies at each genomic position for the two cohorts. The height of each peak corresponds to the percentage of patients in that cohort exhibiting a mutation at the specific position, relative to the Dunlop reference genome (a) or to a genotype-specific consensus sequence (b). Mutations were identified using VarScan with a 20% read frequency threshold. This figure allows visualization of genomic regions more frequently mutated in one cohort compared to the other.

Beyond the global phylogenetic analysis, a more detailed investigation was conducted on the 49 sequences for which the genotype could be determined to identify mutations potentially associated with clinical groups. Variant calling against the Dunlop reference (20% threshold) revealed 425 positions showing at least one divergence (excluding the NCCR). The frequency of each variant within each cohort is shown in Figure 4A. No specific genomic region appeared more prone to genetic diversity in either group. Consensus sequences were also analyzed using the VESPA tool to identify clinically associated patterns, but no significant difference was observed between the HSCT and KTR groups. However, genotype-associated polymorphisms were introduced by using the Dunlop reference (genotype Ia). That is why variants were subsequently called against genotype-specific consensus sequences. This approach reduced the overall number of SNPs detected, yet the distribution of mutations remained comparable between the HSCT and KTR cohorts (Figure 4B).

**Figure 5.**
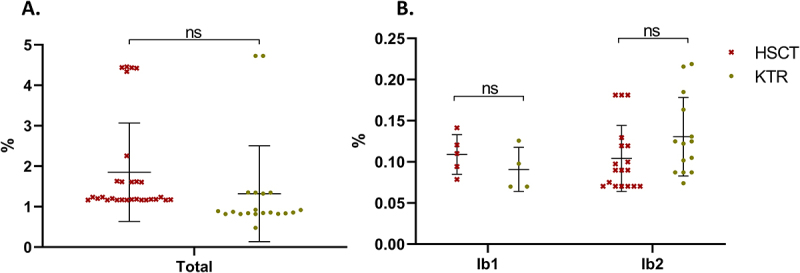
Average BKPyV sequence diversity according to patient cohort (HSCT or KTR). Each point represents the mean phylogenetic distance for a single patient (number of SNPs/number of nucleotides sequenced), calculated from all pairwise comparisons between that patient’s sequence and the sequences of all other patients in the same cohort. a: overall sequence diversity, calculated without considering viral genotype. B: intra-genotype diversity, calculated for subtypes Ib1 and Ib2.

Subsequently, a nucleotide-level diversity analysis was performed, including an assessment of the frequency and nature of these SNPs. Pairwise comparison of NGS-derived BKPyV sequences with an assigned genotype (*n* = 49) revealed a mean phylogenetic distance of 1.63%, with values ranging from 0% to 5.23%; the highest distances corresponded to comparisons between genotype IV and other genotypes. No significant difference was observed between KTR (1.32%) and HSCT (1.85%) (t-test; Figure 5A). The slightly higher mean distance in HSCT was explained by a greater representation of genotype IV in this cohort, a genotype associated with higher inter-genotypic divergence. Intra-subtype analyses restricted to Ib1 and Ib2 confirmed the absence of significant differences between cohorts (Ib1: 0.11% vs. 0.09%; Ib2: 0.10% vs. 0.13%; t-test; Figure 5B).

**Figure 6.**
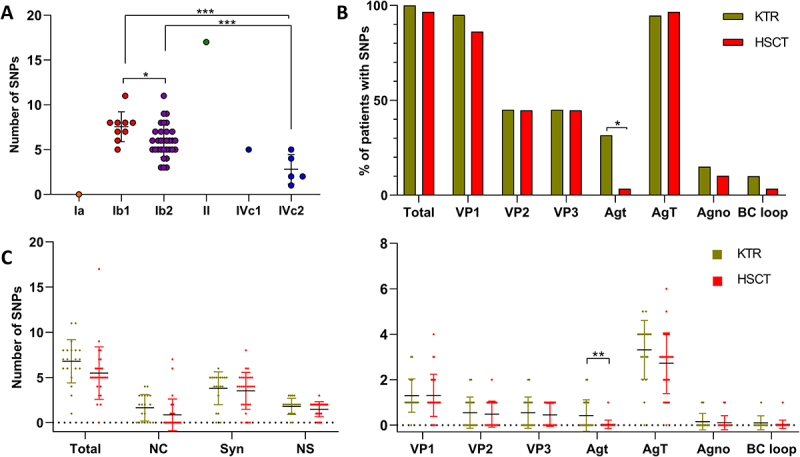
Comparison of SNP distribution between HSCT and KTR patients relative to genotype-specific consensus sequences. A : total number of SNPs per sequence according to viral subtype, reflecting subtype-specific distances to consensus genomes derived from published sequences (Shapiro – Wilk test and t-test : *p < 0.05, ***p < 0.001). B : proportion of patients presenting at least one mutation in each sequenced genomic region (*p < 0.05, Fisher’s exact test). C : quantitative analyses of the mean number of SNPs, performed by mutation type (synonymous, non-synonymous, and non-coding region) and by genomic region (**p < 0.01, t-test). Abbreviations: syn = synonymous; NS = non-synonymous; NC = non-coding region.

The mean number of SNPs relative to the consensus sequence of each genotype varied across subtypes, with mean values of 7.6 for Ib1 and 6 for Ib2, compared with 2.8 for IVc2 (Figure 6A). Mutations predominantly mapped to VP1 and TAg (Figure 6B). The proportion of patients carrying at least one mutation was comparable between cohorts, except for the tAg, which was significantly more frequently mutated in KTR (31.6% vs. 3.4%; *p* = 0.011, Fisher’s exact test). All substitutions identified in tAg were synonymous. Quantitative analysis of the mean number of SNPs similarly showed no cohort-specific difference, except for tAg, which exhibited a higher mutational burden in KTR (0.4 vs. 0.03 SNPs per sequence; *p* = 0.006, t-test; Figure 6C).

An additional objective was to identify parameters associated with severe BKPyV replication within each group – specifically, the occurrence of hemorrhagic cystitis (HC) in HSCT recipients and presumptive BKPyV-associated nephropathy (BKPyVAN) in KTRs. These results are summarized in Tables 2 and 3.

**Table 2.** Clinical and virological characteristics associated with presumptive BKPyVAN in KTR; quantitative data as mean (SEM); qualitative data as n (%). For quantitative variables without normal distribution (Mann-Whitney test), the median [IQR] is also provided.

		No nephropathy (*n* = 46)	Presumptive nephropathy (*n* = 20)	*p-values*
Recipient				
Recipient age (years)		50.44 (1.75)	63.34 (1.9)	**<0.0001**
Recipient sex	Male	32 (69.57%)	16 (80%)	0.549^2^
	Female	14 (30.43%)	4 (20%)	
Recipient weight (kg)		78.94 (2.62)	77.24 (2.65)	0.650^1^
Recipient BMI (body mass index)		26.51 (0.72)	26.65 (0.76)	0.890^1^
Smoking status (Recipient)	Never	22 (48.89%)	8 (40%)	0.537^3^
	Current	8 (17.78%)	6 (30%)	
	Former	15 (33.33%)	6 (30%)	
Pre-transplant dialysis duration (months)		32.66 (6.14) Med: 20 [10.15–37.50]	39.06 (7.96) Med: 31 [23.00–47.50]	0.131^4^
Recipient EBV serostatus	Negative	1 (2.17%)	1 (5%)	0.517^2^
	Positive	45 (97.83%)	19 (95%)	
Recipient CMV serostatus	Negative	21 (45.65%)	10 (50%)	0.955^3^
	Positive	25 (54.35%)	10 (50%)	
Donor-specific antibodies at day 0 (DSA at D0)	No	43 (93.48%)	18 (90%)	0.635^2^
	Yes	3 (6.52%)	2 (10%)	
Pre-transplant residual urine output	<500 mL	17 (40.48%)	5 (33.33%)	0.858^3^
	>500 mL	25 (59.52%)	10 (66.66%)	
Donor				
Donor age (years)		52.09 (1.71)	60.85 (2.51)	**0.006**^**1**^
Donor sex	Female	18 (39.13%)	8 (40%)	1.000^3^
	Male	28 (60.87%)	12 (60%)	
Living donor	No	40 (86.96%)	20 (100%)	0.167^2^
	Yes	6 (13.04%)	0 (0%)	
Transplantation				
Cold ischemia time (minutes)		664.22 (51.78)	804.10 (66.2)	0.103^1^
Induction therapy	Basiliximab	37 (80.43%)	16 (80%)	1.000^2^
	ATG	9 (19.57%)	4 (20%)	
Post-transplant creatinine nadir (µmol/L)		268.04 (32.51) Med = 170.5 [110.00–344.00]	293.15 (48.31) Med = 221.5 [154.25–365.75]	0.364^4^
Maintenance immunosuppressive treatment	Ciclosporine	4 (8.70%)	3 (15%)	0.425^2^
	Tacrolimus	42 (91.30%)	17 (85%)	
Mycophenolate mofetil (MMF) use during maintenance therapy	No	1 (2.17%)	0 (0%)	1.000^2^
	Yes	45 (97.83%)	20 (100%)	
Corticosteroid use during maintenance therapy	No	32 (69.57%)	13 (65%)	0.937^3^
	Yes	14 (30.43%)	7 (35%)	
BKPyV genotype	Ia	4 (9.76%)	1 (5.56%)	0.612^2^
	Ib1	7 (17.07%)	3 (16.67%)	
	Ib2	25 (60.98%)	9 (50%)	
	IV	5 (12.20%)	5 (27.78%)	
Time to BKPyV PCR positivity (days)		102.2 (16.8) Med = 61 [31–140.8]	77.45 (27.6) Med = 34.5 [31.25–46.25]	0.105^4^
Time to BKPyV PCR positivity (days) without outliers (>365 days)		89.5 (11.3) Med = 59 [31-136]	38.61 (6.6) Med = 33 [30-38]	**0.020**^**4**^

^1^Student’s t-test; ^2^ Fisher’s exact test; ^3^ Chi-squared test; ^4^ Mann – Whitney U test (non-normal distribution).

**Table 3.** Clinical and virological characteristics associated with HC in HSCT recipients. Quantitative data as mean (SEM); qualitative data as n (%). For quantitative variables without normal distribution (Mann-Whitney test), the median [IQR] is also provided.

		No BKPyV cystitis (*n* = 21)	BKPyV cystitis (*n* = 14)	*p-values*
	Recipient			
Age (years)		56.33 (3.03) Med = 60.04 [52.16–66.04]	45.25 (5.72) Med = 53.18 [22.40–66.85]	0.14^1^
Sex	Female	9 (42.86%)	3 (21.43%)	0.28^2^
	Male	12 (57.14%)	11 (78.57%)	
Hematologic Malignancy	Acute Lymphoblastic Leukemia	2 (9.52%)	2 (14.29%)	0.92^2^
	Acute Myeloid Leukemia	13 (61.90%)	9 (64.29%)	
	Hodgkin Lymphoma	1 (4.76%)	0 (0%)	
	Non-Hodgkin Lymphoma	2 (9.52%)	0 (0%)	
	Myelofibrosis	1 (4.76%)	1 (7.14%)	
	Myelodysplastic Syndrome	2 (9.52%)	2 (14.29%)	
Transplantation				
Allogeneic Transplant Type	Genotypic Match	1 (4.76%)	0 (0%)	0.22^2^
	Haploidentical	9 (42.86%)	10 (71.43%)	
	Phenotypic Match	11 (52.38%)	4 (28.57%)	
Graft Source	Peripheral blood stem cells	19 (90.48%)	11 (78.57%)	0.37^2^
	Bone Marrow	2 (9.52%)	3 (21.43%)	
Conditioning Regimen	Myeloablative Conditioning	2 (9.52%)	4 (28.57%)	0.19^2^
	Reduced Intensity Conditioning	19 (90.48%)	10 (71.43%)	
Anti-lymphocyte serum (ALS)	No	15 (71.43%)	10 (71.43%)	1.00^2^
	Yes	6 (28.57%)	4 (28.57%)	
Cyclophosphamide Use Post-transplant	No	11 (52.38%)	4 (28.57%)	0.30^2^
	Yes	10 (47.62%)	10 (71.43%)	
Time to BKPyV PCR positivity (days)		32.35 (4.45) Med = 30 [15.5–43.75]	40.1 (10.5) Med = 33 [9.5–44]	0.92^1^
BKPyV Urine Peak (log_10_ copies/mL)		9.19 (0.26) Med = 9.4 [8.70–9.90]	9.43 (0.31) Med = 9.9 [8.90–10.19]	0.36^1^
BKPyV Plasma Peak (log_10_ copies/mL)		2.72 (0.33)	2.95 (0.4)	0.66^3^
BKPyV Genotype	Ia	1 (5%)	0 (0%)	1.00^2^
	Ib1	3 (15%)	2 (16.67%)	
	Ib2	12 (60%)	8 (66.67%)	
	II	1 (5%)	0 (0%)	
	IV	3 (15%)	2 (16.67%)	

^a^Mann – Whitney U test (non-normal distribution); ^2^ Fisher’s exact test; ^3^ Student’s t-test.

Among KTRs, patients with BKPyV replication without nephropathy were significantly younger than those with presumptive BKPyVAN (mean age 50.44 vs 63.34 years; t-test, *p < 0.0001*). Moreover, the first positive BKPyV PCR occurred earlier in the BKPyVAN group (median time to BKPyV PCR positivity (days) without outliers (>365 days): 34.5 vs 61 days; Mann – Whitney U test; *p = 0.02*), suggesting that early post-transplant detection of BKPyV, particularly in older recipients, should raise concern for progression to nephropathy. Conversely, among HSCT recipients, no significant difference was found between patients who developed HC and those who did not, although a trend toward younger age in the HC group was noted (Median age 53.18 vs 60.04 years; Mann – Whitney U test, *p = 0.14*).

Due to the limited number of sequences available (*n* = 51), the statistical power to detect associations between specific viral variants and clinical severity was low in small-sized subgroups. Accordingly, no relevant difference was identified at the sequence level between patients with or without severe BKPyV-related complications.

## Discussion

The study aimed to compare BKPyV replication dynamics, clinical characteristics, and genomic profiles between HSCT recipients and KTR. Two cohorts with similar epidemiological features, including no significant differences in age or sex, allowed a focused comparison based solely on the type of transplant.

### BKPyV kinetics

Contrary to the HSCT cohort, the KTR cohort exhibited a later BKPyV replication dynamic, likely due to differences in transplant type and immunosuppressive treatment. Additionally, the KTR group had a statistically lower peak of BKPyV DNAuria compared to HSCT recipients. This stronger peak in hematology may be explained by more intense immunosuppression in HSCT recipients, who are often subjected to aplasia. Another hypothesis is improved urinary detection if the site of viral replication is the bladder, leading to direct viral shedding in the urine. Additionally, an increased turnover of urothelial cells might enhance viral shedding and thus detection by PCR in urine samples.

The relevance of replication kinetics for clinical outcomes in hematology remains unclear. BKPyV DNAemia sometimes precedes symptoms, suggesting a potential prognostic or predictive role [22–24]; however, its specificity is limited since some patients with DNAemia do not develop cystitis, and others develop cystitis without DNAemia [25,26]. Our study found similar plasma viral load peaks in patients with and without cystitis, contradicting some previous reports [23]. On the contrary, early replication in KTR appears associated with an increased risk of BKPyVAN.

### Clinical risk factors

Some clinical characteristics seem to be potential risk factors of presumptive nephropathy in KTR. Older age appears strongly associated with the development of BKPyV nephropathy. However, the literature on this topic is conflicting. One study identified advanced age (over 50 years) as a risk factor within a validated predictive score [27], while two others found no significant association between age and BKPyV infection or nephropathy [28,29]. A meta-analysis combining five studies concluded that the effect of age is modest: only one study reported a significant association, though a trend is observed in the combined analysis [30]. It is worth noting that most studies compare patients without BKPyV replication to those with replication, whereas our focus is on outcomes exclusively among patients with viral replication. Notably, in our cohort, no presumptive nephropathy case was observed in KTR under 50 years old. This raises questions about immunosenescence, especially since our center does not adjust immunosuppressive regimens based on age. While younger adults tend to control BKPyV replication more effectively, determining the exact age at which risk increases is difficult, as immunosenescence is a gradual process [31]. Still, T-cell immunity begins to decline around 50 years of age [32], which is relevant given its key role in controlling BKPyV. These observations are supported by evidence linking immunosenescence to increased infectious and neoplastic risks [33], and by data showing that lower-intensity regimens are often well tolerated and safer in older KTRs [34].

Among other clinical factors reported in the literature [30], male sex has been described as a risk factor for BKPyVAN. In our study, we observed a similar trend, with a prevalence of 33.3% in replicating men versus 22.2% in replicating women, although the difference was not statistically significant.

In the HSCT group, no significant clinical difference was found between patients with or without HC. However, age under 40 years was identified as a risk factor for HC with an odds ratio of 3.85 in one study [35], which corresponds to the trend observed in our cohort, despite the lack of statistical significance. In addition, there was a trend toward more frequent HC in patients receiving haploidentical transplants, bone marrow grafts, cyclophosphamide, or myeloablative conditioning (i.e. conditions associated with higher cytotoxic exposure). Myeloablative conditioning regimens, with or without cyclophosphamide, have previously been independently associated with BKPyV-associated HC in a study of 409 HSCT patients [36], providing relatively robust evidence compared to the size of our cohort.

### Genomic analysis

The absence of phylogenetic clustering by transplant type and the lack of sequence divergence between groups suggest that host-related factors are more likely to drive the development of BKPyVAN or HC, depending on the type of transplantation.

Regarding genotype-specific associations with complications, no significant link has been demonstrated in the literature between BKPyV genotype and the occurrence of hemorrhagic cystitis [17,18,37], which is consistent with our findings. One study suggested that genotype IVc-2 may confer protection against HC [38]; however, this was not confirmed in our cohort, where four HSCT recipients harbored BKPyV genotype IVc-2, including two with and two without HC.

Genotype IV has been associated with a higher risk of BKPyVAN in KTR [39]. While no significant difference was observed across genotypes in our cohort, genotype IV accounted for a higher proportion of KTR with nephropathy (28%) compared to those without (12%). Notably, 50% (5/10) of patients with genotype IV BKPyV developed presumptive nephropathy, versus 28.8% (17/59) of all genotyped KTR.

Concerning BKPyV genetic diversity, no evidence of co-replications was found in any patient, suggesting minimal intra-host diversity, although the use of amplicon-based PCR could have masked minor variants. Inter-host diversity was also low and primarily reflected differences in genotype distribution. When analyses were restricted to sequences belonging to the same subtype, high homology (~99.9%) was found between sequences, as previously reported for BKPyV subtypes [17,18]. The higher SNPs number observed for Ib1/Ib2 compared with IVc2 likely reflects biases inherent to the subtype-specific reference genomes rather than true biological variability. The only difference observed between cohorts concerned tAg, which exhibited a higher frequency of mutations in KTR. Notably, tAg plays an important role in regulating viral replication [40]. However, all SNPs identified in this region were synonymous, suggesting a limited functional impact, which is consistent with previous reports showing that the J domain of tAg (AA 1–82) is under negative selection and is therefore highly genetically stable – matching our observation [41]. This difference between KTR and HSCT recipients remains intriguing, raising the question of whether these SNPs could play a role, even though no promoter or alternative splice site has been described in this region [42].

This study has several limitations, including its single-center design and relatively small cohort size, as well as the absence of longitudinal sequencing, which limits our ability to assess the temporal dynamics of BKPyV mutations in both transplant groups. However, small cohort sizes are common in the literature on BKPyV in hematopoietic transplantation [43]. In addition, only a subset of patients could be analyzed by NGS due to insufficient sample material or low viral loads, preventing NGS of all samples. Moreover, the NCCR region was excluded because potential rearrangements are difficult to interpret from short-read alignments, and de novo assembly methods can introduce inconsistencies.

## Conclusion

In conclusion, no virological determinant, such as genotype or mutations, appears to be associated with preferential BKPyV replication in KTR versus HSCT recipients. Instead, patient-related factors seem to play a more decisive role in BKPyV replication outcomes in both groups. In KTR, early detection of BKPyV DNAuria may require prompt adjustment of immunosuppressive therapy in older patients who are more susceptible to BKPyVAN. While the role of host immunity in BKPyV replication is relatively well-studied in KTR, further investigations focusing on the immunological determinants of BKPyV replication in HSCT recipients are needed to better identify high-risk individuals.

## Data Availability

The sequences have been deposited in GenBank under accession numbers PV856407 to PV856457. Raw data underlying the figures are available on Figshare [44] at https://doi.org/10.6084/m9.figshare.30802961, except for the clinical data, which may be indirectly identifying and are therefore provided in aggregated form in Tables 1 to 3 without individual-level information.
